# Excessive conjunctival and scleral retraction during trabeculectomy: an unusual intraoperative complication

**DOI:** 10.22336/rjo.2023.17

**Published:** 2023

**Authors:** Fatih Özcura, Alpaslan Koç, Saadet Gültekin Irgat

**Affiliations:** * Department of Ophthalmology, Kutahya Health Sciences University School of Medicine, Kutahya, Turkey

**Keywords:** conjunctival retraction, conjunctival autograft transplantation, scleral retraction, spondyloarthropathy, trabeculectomy

## Abstract

**Aim:** Trabeculectomy is the gold standard surgery for achieving target intraocular pressure (IOP) in glaucoma. Besides the efficiency of trabeculectomy, intraoperative or postoperative complications such as, suprachoroidal hemorrhage, vitreous loss, malignant glaucoma, flat anterior chamber, hypotony, choroidal detachment, endophthalmitis, are also quite important. We present the management of excessive conjunctival and scleral retraction during trabeculectomy: an unusual intraoperative complication.

**Case report:** A 66-year-old woman was referred to our glaucoma unit with progression of primary open angle glaucoma. No known systemic disease was observed in her history except hypertension. The best-corrected visual acuity was 20/ 63 in the right eye and 20/ 20 in the left eye. IOP was 27 mmHg and 19 mmHg (with bimatoprost timolol fixed combination and brimonidine tartrate) in the right and left eyes, respectively. We planned trabeculectomy with mitomycin C for the right eye of the patient. Excessive conjunctival and scleral retraction occurred during surgery. Autograft conjunctival tissue was prepared to cover for bare sclera area. No complications were observed in postoperative period. Seronegative spondyloarthropathy (HLA-B27-negative) was diagnosed postoperatively as a result of consultations.

**Discussion:** Conjunctival retraction is observed as a postoperative complication after trabeculectomy. Postoperative conjunctival retraction can cause bleb leakage and hypotony, as well as predispose to infection. Nowadays, micro invasive glaucoma surgery (MIGS) is gaining popularity, especially because of its reduced complication rate compared to trabeculectomy. However, considering the IOP reduction rates, MIGS has been indicated in mild and moderate glaucoma.

**Conclusions:** We presented the management of excessive conjunctival and scleral retraction during trabeculectomy, which has not been reported earlier. Conjunctival autograft transplantation is useful to manage this complication.

## Introduction

Trabeculectomy is considered the gold standard surgery for achieving target intraocular pressure (IOP) in glaucoma. It is a very effective IOP lowering procedure than the current micro invasive glaucoma surgeries (MIGS). Besides the efficiency of trabeculectomy, quite important intraoperative or postoperative complications such as, suprachoroidal hemorrhage, vitreous loss, malignant glaucoma, flat anterior chamber, hypotony, choroidal detachment, endophthalmitis, also exist [**1**]. We presented the management of excessive conjunctival and scleral retraction during trabeculectomy: an unusual intraoperative complication. To our best knowledge, this is an unusual intraoperative complication that has not been reported in literature. Kutahya Health Sciences University Clinical Research Ethics Committee approved this report. The patient also signed an informed consent to authorize the publication of this case report.

## Case report

A 66-year-old woman was referred to our glaucoma unit with progression of primary open angle glaucoma. No known systemic disease in her history was observed, except hypertension. The best-corrected visual acuity was 20/ 63 in the right eye and 20/ 20 in the left eye. IOP was 27 mmHg and 19 mmHg (with bimatoprost timolol fixed combination and brimonidine tartrate) in the right and left eyes, respectively. Ocular examination revealed bilateral pseudophakia, cup-to-disk ratio was 0.95 and 0.8 in the right and left eyes, respectively. Visual field revealed superior and inferior arcuate scotoma in the right eye and inferior arcuate scotoma in the left eye, retinal nerve fiber layer thickness also concordant with the visual field. Anterior chamber depth was 3.35 mm and 3.43 mm, anterior chamber angle was 39.1° and 43.3°, central corneal thickness was 607 μm and 570 μm in the right and left eyes, respectively.

**Fig. 1:**
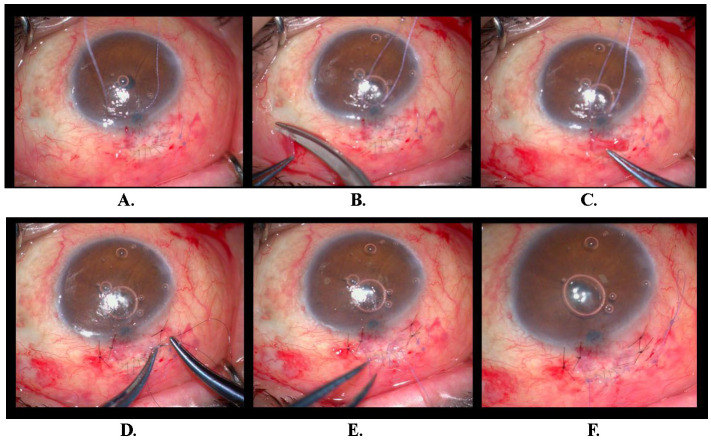
Closing the retracted conjunctiva by two 8-0 vicryl sutures and suturing the scleral flap **(A)**, preparing the autograft conjunctival tissue **(B)**, retraction of the autograft conjunctival tissue **(C)**, fixation of autograft conjunctiva by atraumatic monofilament 10-0 nylon suture **(D)**, continuous suturing superior margin of autograft conjunctival tissue to the free conjunctival edge **(E)**, end of the surgery **(F)**

**Fig. 2:**
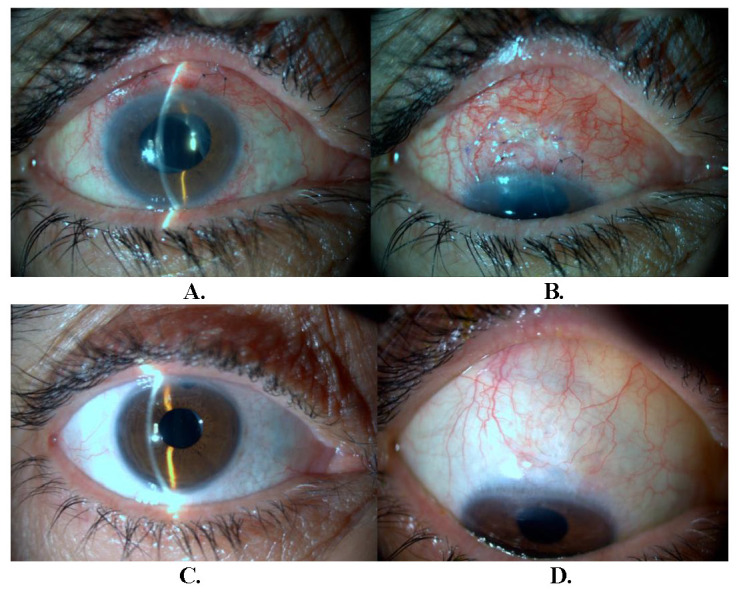
Postoperative photographs of the patient (**A** and **B** 1 week, **C** and **D** 6 months) after the surgery

We planned trabeculectomy with mitomycin C for right eye of the patient. The conjunctiva was opened with a fornix-based incision and a trapezoidal scleral flap was formed on the superior quadrant of the eye. Excessive conjunctival and scleral retraction occurred during surgery. The scleral flap could be closed with a 9 interrupted monofilament 10-0 nylon suture. The retracted conjunctiva was closed by two 8-0 vicryl sutures. Autograft conjunctival tissue was prepared to cover for bare sclera area. The same retraction was observed in autograft conjunctival tissue, but the area of bare sclera could be transplanted and fixed by an atraumatic monofilament 10-0 nylon suture. The superior margin of autograft conjunctival tissue was sutured with 8-0 vicryl continuous suture to the free conjunctival edge (**Fig. 1**). No complications were observed in the postoperative period and IOP ranged from 12 to 18 mmHg during the 20-month follow-up (**Fig. 2**). Seronegative spondyloarthropathy (HLA-B27-negative) was diagnosed postoperatively as a result of consultations completed in terms of underlying possible systemic diseases.

## Discussion

Conjunctival retraction is observed as a postoperative complication of trabeculectomy. Postoperative conjunctival retraction can cause bleb leakage, hypotony, and predisposition to infection [**2**]. Prevention of wound leakage and hypotony is very important in trabeculectomy. Wound leaks can lead to flattened bleb morphology, hypotony and increase the risk of trabeculectomy failure [**3**]. Postoperative leakage did not occur in our case.

 In the search for the perfect glaucoma surgery, a lot of innovations and devices have come into the market. These are collectively labeled as MIGS and are ab-interno glaucoma procedures often sparing the conjunctiva, and thought to be less aggressive than the trabeculectomy [**4**]. Nowadays, MIGS are gaining popularity, especially because of its reduced complication rate compared to trabeculectomy. However, considering the IOP reduction rates, MIGS has indications in mild and moderate glaucoma [**5**]. Trabeculectomy was preferred in our case because of an advanced glaucoma [**6**] with no ocular surface pathology or known connective tissue disorders.

A diagnosis of seronegative spondyloarthropathy was made after postoperative discussions. The most common ocular manifestation of seronegative spondyloarthropathies is anterior uveitis. Other non-uveitis manifestations of seronegative spondyloarthropathies include episcleritis, scleritis, keratitis, orbital inflammation, and optic neuropathy [**6**]. Our patient had no history or signs of uveitis during the ocular examinations. The main pharmacological therapy is a maximum dose of a non-steroidal anti-inflammatory drug (NSAID). In our case, topical and systemic NSAID was started by the rheumatologist.

The pathophysiology of seronegative spondyloarthropathy is not completely and clearly known. Unusual wound healing responses could result in spondyloarthropathies, because chronic inflammation is an essential pathway underlying the pathogenesis [**7**]. Similarly, unusual complications can also occur during surgeries.

In our case, we irrigated with plenty of water after making an intraoperative MMC application for 2 minutes. The MMC application might have provoked the retraction of the conjunctiva and sclera. If the trabeculectomy is performed in patients with preoperative seronegative spondyloarthropathy without MMC application, then the retraction that might have occurred is prevented.

Herein, we presented the management of excessive conjunctival and scleral retraction during trabeculectomy that has not been reported before. Conjunctival autograft transplantation is useful in the management of this complication. Also, we observed that there is a serious retraction in the autograft tissue we received. When such a complication is encountered, we believe that dimensions larger than the normal autograft tissue prepared will make the management even easier. Ab interno glaucoma surgeries or cyclodestructive procedures may be considered in cases with known spondyloarthropathy or similar diseases before surgery. However, these procedures may not be suitable in some seronegative spondyloarthropathy cases. In these cases, ab externo surgery may be the only option. Limbal based conjunctival incision should be preferred in case ab externo surgery is to be performed.

## Conclusion

Conjunctival autograft transplantation is useful to manage this complication. Ab interno glaucoma surgeries or cyclodestructive procedures may be considered in cases with known spondyloarthropathy or similar disease before surgery. Limbal based conjunctival incision should be preferred if it will be performed ab externo surgery.

**Conflict of Interest statement**

The author(s) declare no potential conflicts of interest with respect to the research, authorship, and/ or publication of this article.

**Informed Consent and Human and Animal Rights statement**

Informed consent has been obtained from the individual included in this study.

**Authorization for the use of human subjects**

Ethical approval: The research related to human use complies with all the relevant national regulations, institutional policies, is in accordance with the tenets of the Helsinki Declaration, and has been approved by Kutahya Health Sciences University Clinical Research Ethics Committee, Kutahya, Turkey.

**Acknowledgements**

None.

**Sources of Funding**

The author(s) received no financial support for the research, authorship, and/ or publication of this article.

**Disclosures**

None.
